# Low vitamin D levels are associated with cognitive impairment in patients with Hashimoto thyroiditis

**DOI:** 10.1186/s12902-018-0314-7

**Published:** 2018-11-26

**Authors:** Jun Xu, Xiang-yun Zhu, Hui Sun, Xiao-qin Xu, Song-ao Xu, Yuan Suo, Li-jun Cao, Qiang Zhou, Hui-jie Yu, Wei-zhong Cao

**Affiliations:** 1 Department of Emergency, First Affiliated Hospital of Jiaxing University, Jiaxing, Zhejiang Province China; 2 Department of Endocrinology, First Affiliated Hospital of Jiaxing University, Jiaxing, Zhejiang Province China

**Keywords:** Hashimoto’s thyroiditis, Vitamin, Cognitive impairment, Montreal cognitive assessment

## Abstract

**Background:**

Cognitive impairment is commonly observed in patients with Hashimoto thyroiditis (HT). Low levels of vitamin D have been correlated with cognitive impairment in non-HT population. We examined the association of vitamin D levels with cognitive impairment in patients with HT.

**Methods:**

We recruited 194 patients with HT and 200 healthy volunteers. Levels of serum 25-hydroxyvitamin D (25(OH)D) were measured using a competitive protein-binding assay. Cognitive funtion was assessed using Montreal Cognitive Assessment score (MoCA). Subjects with a MoCA scores < 26 are considered as having mild cognitive impairment (MCI). Multivariate analysis was performed using logistic regression models.

**Results:**

Fifty-five HT patients (28.4%) were diagnosed as having MCI. Patients with MCI had significantly lower 25(OH)D levels when compared with patients without MCI (33.9 ± 6.2 vs. 44.3 ± 9.6 nmol/L, *P* < 0.001). Significant differences in 25(OH)D quartiles of HT patients were observed between the patients with MCI and the patients without MCI (*P* < 0.001). In multivariate analyses, serum 25(OH)D levels (≤ 34.0 and ≥ 47.1 nmol/L) were significantly associated with cognitive impairment in patients with HT (OR 6.279, 95% CI 2.673–14.834, *P* < 0.001; OR 0.061, 95% CI 0.008–0.491, *P* = 0.009, respectively).

**Conclusion:**

Our results demonstrate an important association between serum vitamin D levels and cognitive impairment in patients with HT.

## Background

Hashimoto thyroiditis (HT) is a common chronic autoimmune disease of the thyroid gland, characterized by painless goiter and elevated thyroid antibodies. A growing number of studies suggest cognitive impairment in patients with HT, independently of thyroid function disorders [1, 2]. Cognitive impairment has been associated with increased risk of depression and impaired activities of daily living in patients with chronic diseases [3, 4]. Therefore, it is important to identify risk factors for the presence of cognitive impairment in patients with HT.

In addition to its well-known significance in the regulation of calcium-phosphorus metabolism, vitamin D may have neuroprotective properties by suppressing inflammation and oxidative stress [5–7]. Vitamin D receptors and vitamin D activating enzyme 1α-hydroxylase are broadly present in the hippocampus, hypothalamus, cortex and subcortex, the regions essential for cognition [8, 9]. Emerging clinical studies suggest an association between low vitamin D levels and cognitive impairment in adults and non-HT patients with chronic kidney disease, type 2 diabetes, as well as Alzheimer’s disease [10–14].

Hypovitaminosis D (vitamin D insufficiency and deficiency) is common among patients with HT [15–17]. Up to now, however, no study has explored the possible association between vitamin D levels and cognitive impairment in patients with HT. Given the involvement of vitamin D in cognitive impairment among non-HT subjects and the high prevalence of hypovitaminosis D in patients with HT, whether vitamin D levels are related to cognitive impairment in HT patients was examined.

## Methods

### Study sample

Of 212 patients diagnosed as HT were consecutively recruited from the First Affiliated Hospital of Jiaxing University between 16 March 2014 and 24 February 2017. Diagnosis of HT was based on positive anti-thyroid peroxidase antibodies (TPOAbs) and/or anti-thyroglobulin antibodies (TgAbs), associated with a ultrasound patterns suggestive of HT. Eligibility criteria included: (1) Chinese ethnicity; (2) age from 18 to 60 years; (3) stable L-thyroxine treatment; (4) harboring the willingness to give informed consent. Exclusion criteria were: (1) patients with a history of other endocrine diseases; (2) patients with a history of cognitive or psychiatric disorders; (3) patients with a history of osteoporosis; (4) patients receiving vitamin D replacement therapy. Meanwhile, 200 healthy volunteers without any euthyroid, neurological or psychiatric diseases, were recruited from a health survey of the same regions.

### Clinical variables

Demographic and clinical variables were obtained from participant report and electronic medical records. Demographic inclued age, sex, body mass index (BMI) and education. BMI was calculated as weight (kg)/squared height (m^2^). A fasting morning venous blood sample was obtained from each participant. Levels of serum thyroid-stimulating hormone (TSH), free-triiodothyronine (FT3), free thyroxine (FT4), TgAbs, and TPOAbs were determined with automated immuno chemiluminescent assay (ICMA) kits (Abbott Laboratories, IL, USA). Levels of serum 25-hydroxyvitamin D (25(OH)D) were determined using a competitive protein-binding assay (Roche Diagnostics, Mannheim, Germany). The inter-assay variation coefficient for 25(OH)D measurement was 8.5%. Serum 25(OH)D levels in HT patients were divided into four quartiles (≤ 34.0, 34.1–40.0, 40.1–47.0 and ≥ 47.1 nmol/L), as the raw data of 25(OH)D were skewed. The median 25-hydroxyvitamin D values for all quartiles were 30.8, 36.6, 43.7 and 53.1 nmol/L, respectively.

### Neuropsychological testing

Cognitive funtion was assessed using Montreal Cognitive Assessment score (MoCA), a simple cognitive screening tool with superior sensitivity [18]. The MoCA is a one-page 30-point test that can be administered in 10 min. It covers important cognitive domains, including short-term memory, visuospatial abilities, executive functions, attention, concentration, working memory, and language, together with orientation to time and place. Based on the MoCA scoring system, patients were divided into two groups: the patients who had MoCA scores < 26 were selected as mild cognitive impairment (MCI) group, and the other patients who had MoCA scores of 26 or greater were selected as the group without MCI. These evaluations were administered by the same experienced psychologist who was blind to the laboratory results of HT patients.

### Statistical analysis

Data were presented as number (percentage) for categorical variables, mean ± standard deviation (SD) for normally distributed variables, and medians (25th, 75th percentiles) for non-normally distributed variables. Comparisons between the groups were conducted using the *χ*^*2*^ test, Fisher’s exact test, Student *t* test, and Mann–Whitney *U* test, as appropriate. Binary logistic regression including age, sex, and the factors with *P* < 0.10 in the univariate analysis, was performed to examine significant risk factors for cognitive impairment in HT patients. The abnormally distributed parameters were log-transformed for satisfying the log-linearity assumption. The results were presented as odds ratios (OR) with corresponding 95% confidence intervals (CI). All Statistical analysis were performed by using SPSS 22.0 (Chicago, IL). Significance level was defined as *P* < 0.05.

## Results

### Baseline characteristics of study samples

Of 212 patients with HT, 18 were excluded from this analysis: 2 with a history of dementia, 6 taking vitamin D replacement therapy, and 10 who refused to participate in this study. There were no significant differences in age and sex between our study cohort (*N* = 194) and those excluded. Of 194 participants, 158 were male (81.4%) and their mean (SD) age was 49.4 (9.8) years. The patients in this study did not differ from the controls in terms of age, sex, BMI, education, as well as levels of TSH, FT3, and FT4 (all *P* > 0.05) (Table 1).

**Table 1:** Characteristics of HT patients and controls

Characteristics	Patients with HT	Controls	*P*
Number of patients	194	200	
Age (years), mean ± (SD)	49.4 ± 9.8	48.6 ± 9.1	0.352
Sex (female/male)	36/158	43/157	0.466
Educaton (years), median (IQR)	11 (8–14)	12 (9–14)	0.255
BMI (kg/m2), mean ± (SD)	24.2 ± 3.2	24.5 ± 3.3	0.437
TSH (IU/mL), mean ± (SD)	2.2 ± 0.9	2.0 ± 0.8	0.169
FT3 (pmol/L), mean ± (SD)	4.8 ± 1.1	4.6 ± 1.2	0.229
FT4 (pmol/L), mean ± (SD)	18.1 ± 4.4	18.6 ± 4.7	0.254
25(OH)D (nmol/L), median (IQR)	40.4 (34.3–46.9)	58.3 (52.1–64.8)	< 0.001

Data are expressed as number (percentage) or means (± SD) or medians (IQR). Abbreviations: *HT* Hashimoto thyroiditis, *BMI* Body mass index, *TSH* Thyroid-stimulating hormone, *FT3* Free-triiodothyronine, *FT4* Free thyroxine, *25(OH)D* 25-hydroxyvitamin D

### Univariate associations

**Fig. 1:**
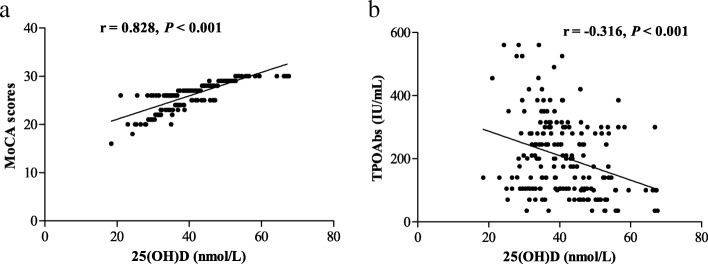
The correlation between serum 25(OH)D levels and **a** MoCA scores as well as TPOAbs levels. Abbreviations: MoCA, Montreal Cognitive Assessment score; 25(OH)D, 25-hydroxyvitamin D; **b** TPOAbs, anti-thyroid peroxidase antibodies

Of the 194 patients who formed the study sample, 55 (28.4%, 42 female, 13 male) were diagnosed with MCI. Serum levels of 25(OH)D were markedly lower in patients with HT than in healthy controls (40.4 (34.3–46.9) vs. 58.3 (52.1–64.8) nmol/L, *P* < 0.001). There was a postive correlation between 25(OH)D levels and MoCA scores (*r* = 0.828, *P* < 0.001; Fig. 1a). Compared with patients without MCI, patients with MCI had significantly lower 25(OH)D levels (33.9 ± 6.2 vs. 44.3 ± 9.6 nmol/L, *P* < 0. 001), while serum TSH, FT3, FT4, and antibodies levels were not different. 25(OH)D levels were inversely correlated with TPOAbs levels (*r* = − 0.316, *P* < 0.001; Fig. 1b). No correlation was observed between 25(OH)D levels and age, sex, BMI, education, as well as levels of TSH, FT3, FT4, and TgAbs (all *P* > 0.05). Significant differences in 25(OH)D quartiles of HT patients were detected between the patients with MCI and the patients without MCI (*P* < 0.001) (Table 2).

**Table 2:** Patient characteristics stratified by MCI

Characteristics	Patients with MCI	Patients without MCI	*P*
Number of patients	55	139	
Age (years), mean ± (SD)	46.7 ± 9.0	50.5 ± 9.9	0.014
Sex (female/male)	13/42	23/116	0.252
Educaton (years), median (IQR)	13 (8–17)	11 (8–14)	0.074
BMI (kg/m2), mean ± (SD)	23.6 ± 3.1	24.5 ± 3.2	0.092
TSH (IU/mL), mean ± (SD)	2.3 ± 0.8	2.1 ± 0.9	0.336
FT3 (pmol/L), mean ± (SD)	4.7 ± 1.0	4.8 ± 1.1	0.541
FT4 (pmol/L), mean ± (SD)	17.9 ± 3.9	18.2 ± 4.6	0.710
TPOAbs (IU/mL), median (IQR)	210.1 (105.0–280.8)	175.4 (100.6–280.6)	0.244
TgAbs (IU/mL), median (IQR)	221.2 (201.5–282.3)	213.5 (190.3–248.5)	0.135
25(OH)D (nmol/L), mean ± (SD)	33.9 ± 6.2	44.3 ± 9.6	< 0.001
25(OH)D, No. (%)			< 0.001
Quartile 1 (30.8 nmol/L)^a^	29 (52.7)	20 (14.4)	< 0.001
Quartile 2 (36.6 nmol/L)^a^	14 (25.5)	31 (23.3)	0.639
Quartile 3 (43.7 nmol/L)^a^	10 (18.2)	40 (28.8)	0.128
Quartile 4 (53.1 nmol/L)^a^	2 (3.6)	48 (34.5)	< 0.001

Data are expressed as number (percentage) or means (± SD) or medians (IQR). Abbreviations: *MCI* Mild cognitive impairment, *BMI* Body mass index, *TSH* Thyroid-stimulating hormone, *FT3* Free-triiodothyronine, *FT4* Free thyroxine, *TPOAbs* Anti-thyroid peroxidase antibodies, *TgAbs* Antithyroglobuline antibodies, *25(OH)D* 25-hydroxyvitamin D

^a^The median 25-hydroxyvitamin D values for each quartile

### Multivariate regressions

With all HT patients taken as a whole, quartile 2 and quartile 3 taken as the references used for serum 25(OH)D levels, and cognitive impairment taken as a dependent variable in the logistic analysis, serum 25(OH)D levels (≤ 34.0 and ≥ 47.1 nmol/L) were significantly associated with cognitive impairment in patients with HT (OR 6.279, 95% CI 2.673–14.834, *P* < 0.001; OR 0.061, 95% CI 0.008–0.491, *P* = 0.009, respectively) (Table 3).

**Table 3:** Characteristics associated with MCI in patients with HT

Characteristics	OR (95% CI)	*P*
25(OH)D		
Quartile 1	6.279 (2.673–14.834)	< 0.001
Quartile 4	0.061 (0.008–0.491)	0.009
Sex	1.153 (0.487–2.728)	0.746
Age	1.022 (0.978–1.068)	0.323
Education	1.040 (0.982–1.102)	0.176
BMI	0.909 (0.810–1.020)	0.103

Abbreviations: *MCI* Mild cognitive impairment, *HT* Hashimoto thyroiditis, *25(OH)D* 25-hydroxyvitamin D, *BMI* Body mass index

## Discussion

To our knowledge, this is the first study examining the possible association of serum level of vitamin D with cognitive impairment in patients with HT. Our results suggest that serum vitamin D levels were significantly associated with cognitive impairment in HT patients, which is similar to earlier studies in adults and non-HT patients with chronic kidney disease, type 2 diabetes, as well as Alzheimer’s disease [10–14]. Further studies shuold be encouraged to examine the preventive and therapeutic effects of vitamin D on cognitive impairment in patients with HT. Moreover, Yasmeh et al. reported that 25(OH)D levels for the HT and controls were significantly different in females but not in males. But no correlation was observed between 25(OH)D levels and sex in the current study. Further studies are needed to determine the sex difference of 25(OH)D levels in HT patients.

Vitamin D receptors and vitamin D activating enzyme 1α-hydroxylase are broadly expressed in the hippocampus, hypothalamus, cortex and subcortex, the regions essential for cognitive function [8, 9]. Additionally, emerging experimental studies suggest an important neuroprotective role of vitamin D by mediating expression of neurotransmitters, improving neurogenesis, and preventing amyloid-β accumulation [19–21]. Studies on vitamin D receptors knockout mice have shown that hypovitaminosis D may play a role in behavioural, motor and sensory deficits, all of which can contribute to cognitive impairment [22–24]. In line with these results, clinical evidence shows that low levels of vitamin D is associated with cognitive impairment.

The exact role of vitamin D in the pathophysiology of cognitive impairment in HT patients is not yet clear. A possible explanation is the effect of vitamin D on inflammatory cytokines. Previous studies showed an increased cytokine production, including interleukin-1β (IL-1β), interleukin-6 (IL-6), monocyte chemoattractant protein-1 (MCP-1), and tumor necrosis factor alpha (TNF-α), in HT patients [25, 26]. Vitamin D has been involved in modulating the secretion of cytokines, including TNF-α, IL-6, IL-1β, and MCP-1 [27–29]. Numerous studies support the notion that inflammation, characterized by elevated cytokines, play a pivotal role in the pathogenesis of cognitive impairment [30, 31]. Therefore, these results suggest that vitamin D might play a key role in cognitive impairment of patients with HT.

Several limitations of the study should be presented. First, the limited number of HT patients reduced the statistical power of the study. Second, the time variation of vitamin D levels makes it preferable to perform the measurements on the same day. Third, the cross-sectional nature of the study precludes any conclusions on the possbile causal relationship between vitamin D levels and cognitive impairment in patients with HT. Forth, the study subjects came from only one clinic, which limited the generalization of the findings of the study. Fifth, it was not possible totest the correlation between 25(OH)D levels and MoCA scores in the control cohort due to lack of MoCA scores of healthy volunteers. Finally, the effects of dietary intake and lifestyle manners on vitamin D levels, were not considered in this study.

## Conclusion

Our findings indicate a significant relationship of serum vitamin D levels with cognitive impairment in patients with HT. Further studies should be encouraged to replicate our findings in larger populations, using a comprehensive battery of tests reduced to selected cognitive domains.

## Abbreviations

25(OH)D: 25-hydroxyvitamin D
FT3: Free-triiodothyronine
FT4: Free thyroxine
HT: Hashimoto thyroiditis
IL-1β: interleukin-1β
IL-6: interleukin-6
MCI: Mild cognitive impairment
MCP-1: Monocyte chemoattractant protein-1
MoCA: Montreal Cognitive Assessment score
TgAbs: Anti-thyroglobulin antibodies
TNF-α: Tumor necrosis factor alpha
TPOAbs: Anti-thyroid peroxidase antibodies
TSH: Thyroid-stimulating hormone
